# Automating ALCHEMI at the nano-scale using software compatible with PC-controlled transmission electron microscopy

**DOI:** 10.1107/S1600576722003818

**Published:** 2022-05-25

**Authors:** Akimitsu Ishizuka, Masahiro Ohtsuka, Shunsuke Muto

**Affiliations:** a Department of Materials Physics, Graduate School of Engineering, Nagoya University, Chikusa-ku, Nagoya, 464-8603, Japan; b HREM Research Inc., 14–48 Matsukazedai, Higashimastuyama, 355-0055, Japan; c Electron Nanoscopy Section, Advanced Measurement Technology Center, Institute of Materials and Systems for Sustainability, Nagoya University, Chikusa-ku, Nagoya, 464-8603, Japan

**Keywords:** electron channeling, site-selective microanalysis, nano-scale analysis, automated probe shift correction, energy-dispersive X-ray spectroscopy, electron energy-loss spectroscopy

## Abstract

Atom location by channeling-enhanced microanalysis (ALCHEMI) is a technique to obtain atom-site-specific information on constituent elements in a crystalline sample by acquiring a set of core electron transition spectra while tilting the incident beam. This methodology has been extended to a more quantitative technique called high-angular-resolution electron-channeled X-ray/electron spectroscopy (HARECXS/HARECES). There is a growing demand for analyzing smaller areas, such as small particles and multilayers. However, the minimum size of a region of interest probed by the present hardware-assisted automated HARECXS/HARECES scheme is limited to no smaller than 1 µm, not only by the size of the electron probe and its convergence angle but also by the movement of the probe position associated with the beam tilt due to aberrations of the hardware system. Herein, *QED* (quantitative electron diffraction), a commercial plug-in working on an integrated software platform, *Gatan Microscopy Suite*, was modified to enable the calibration and control of the probe to resolve the aforementioned limitation. In addition, a more sophisticated scheme for *QED* was developed to realize the ALCHEMI method for energy-dispersive X-ray spectroscopy, electron energy-loss spectroscopy or both concurrently. This allows access to ALCHEMI and its derivative methods, automatically executed with any type of current PC-controlled commercial microscope on an area as small as 30 nm, without modifying the hardware system.

## Abstract 2

A software-control system has been developed that enables access to site-selective energy-dispersive X-ray spectroscopy/electron energy-loss spectroscopy measurements from a sub-micrometre area by automatically and concurrently tilting the beam and acquiring spectra.

## 1. Introduction

Transmission electron microscopy (TEM) has been recognized as an indispensable analytical tool for current research and development covering a wide range of material research fields. In particular, it is important to know which site impurity or intentionally doped element occupies one or more crystallographically inequivalent sites among the host element sites, and how they change the macroscopic chemical/electronic properties of the material. A current trend to tackle such problems is to directly observe and analyze the impurities using an electron probe focused to a few sub-ångtröms in diameter, thereby exploiting not only atomic-column-resolution scanning images but also spectroscopic data localized at each atomic column. Aberration-correction (AC) technologies are common in the electron microscopy field, with significant progress being achieved in scanning TEM (STEM). It is, however, not always possible to access site-specific information, particularly when it comes to quantitative analysis, by atomic column-by-column AC-STEM analysis when the sample is thicker than a few tens of atomic layers or the impurity concentration is lower than a few atomic percentages.

In general, electrons that enter a periodic potential field, such as in a crystalline sample, propagate inside the crystal as Bloch waves, and the electron wavefunctions spread over the outer areas from the directly illuminated area with increasing sample thickness. This delocalization effect is one of the main factors that render quantitative atomic column-by-column AC-STEM analysis difficult. In this respect, an alternative experimental scheme had been proposed, taking advantage of the fact that the excitation weight of each Bloch wave with different symmetry varies with the change in the diffraction condition, each propagating along a different set of atomic columns or planes (electron-channeling effects). The scheme was originally called atom location by channeling-enhanced microanalysis (ALCHEMI). ALCHEMI is a technique for analyzing the site occupancies of the constituent elements in a crystalline sample from the variations of spectral intensity, using energy-dispersive X-ray spectroscopy (EDX) (Taftø & Spence, 1982 ▸) and electron energy-loss spectroscopy (EELS) (Taftø & Krivanek, 1982 ▸), taken at several selected diffraction conditions. Here, the diffraction conditions are changed by tilting the sample using the sample holder. However, tilting the sample with the holder introduces appreciable sample drift, which cannot be ignored when analyzing a small area of the sample.

The ALCHEMI method has been refined to more sophisticated forms, called high-angular-resolution electron-channeled X-ray or electron spectroscopy (HARECXS or HARECES, respectively), where the incident electron beam is consecutively tilted to excite the systematic reflections associated with the atomic planes stacked at different inter-planar intervals (Matsumura *et al.*, 1999 ▸; Yasuda *et al.*, 2007 ▸; Tatsumi & Muto, 2009 ▸). Furthermore, the present authors’ group has extended the HARECXS method to a 2D beam-rocking scheme, taking advantage of the digital beam control system of their current scanning transmission electron microscope. This provides a simple and highly quantitative atom-location method to access information on not only point defects, such as substitutional dopants and impurities, but also vacancies (Ohtsuka *et al.*, 2021 ▸), interstitials and extended defects, if diffraction-based methods such as synchrotron X-ray and neutron diffraction are not easy to access. A video clip demonstration of the experimental methodology and analysis protocol of 2D-HARECXS has been presented earlier (Ohtsuka & Muto, 2021 ▸).

Normally, ALCHEMI and its derivative techniques require a single-crystalline area, and the incident electron beam illuminates a small area at a convergence semi-angle smaller than a few milliradians. The requirement to fix the illuminated probe position (pivot point) during the beam rocking by a few degrees or more limits the illuminated area to no smaller than 1 µm in diameter because of the aberrations of the illuminating lens system. This limitation makes it challenging to apply this method to nanoparticles and local structures near an interface. In the current advanced STEM systems, a nano-beam mode is available or optionally implemented, which allows the nearly parallel incident electron probe to be focused down to as small as a few nanometres in diameter. However, the illuminated probe moves over an area that is larger than the probe diameter when beam rocking is implemented using the present hardware system, which should be compensated for to keep the illuminated probe fixed at the area of interest (Muto & Ohtsuka, 2017 ▸).

In this study, we developed a software script for the ALCHEMI mode in *QED* (quantitative electron diffraction, which controls the beam-rocking function with the beam shift compensated for and synchronizes the beam tilt with the camera, EDX detector and/or EELS detector; HREM Research, 2011 ▸). *QED* is a commercial plug-in working on an integrated software platform, *Gatan Microscopy Suite* (*GMS*) [also known as *DigitalMicrograph* (*DM*), an integrated measurement/analysis platform commonly used in the TEM research community; https://www.gatan.com/products/tem-analysis/gatan-microscopy-suite-software]. The ALCHEMI mode of *QED* with the developed script lifts the beam size limitation in the conventional hardware control scheme described earlier. Test measurements using a nano-beam are demonstrated for obtaining EDX/EELS ionization-channeling patterns from sub-micrometre areas.

## 2. Concept of software beam control for ALCHEMI

### 2.1. Current data structure of ALCHEMI

A scanning transmission electron microscope can collect spectral signals (*e.g.* by EDX or EELS detector) by scanning a focused small probe on a specified area of a specimen as a function of the probe position, the scheme of which is called spectral imaging. The data set obtained has a two-, three- or four-dimensional structure and consists of 1D (line scan) or 2D spatial coordinates (*x*, *y*) and the 1D detector channels *E_n_* (EDX, EELS, cathodoluminescence *etc*.; 3D data cube) or 2D coordinates in reciprocal space ( 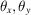 ) (diffraction imaging; 4D-STEM).

**Figure 1:**
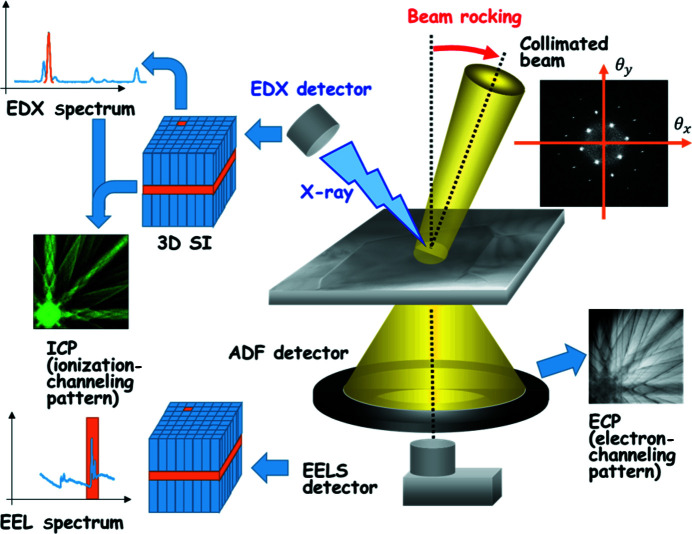
Schematic diagram showing HARECXS/HARECES experimental setups based on the incident-beam-rocking scheme.

On the other hand, when the spectral signals are recorded by rocking a quasi-parallel incident electron beam with the beam pivot point fixed on a crystalline specimen, another type of information can be obtained, where the spectral intensity of a constituent element as a function of the beam-tilt angle exhibits a characteristic profile depending on the crystallographic site that the element occupies as a result of the electron-channeling effects. The data structure is 2D (1D tilt along the systematic row of Bragg reflections) or 3D (more general 2D tilt), consisting of the tilt angle and detector channel coordinates ( 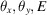 ), as shown in Fig. 1 ▸. The resulting image is called an electron-channeling pattern (ECP) and is the counterpart of the annular dark field (ADF) STEM image, the signal from the ADF detector, as a function of the tilting angle ( 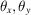 ) in the 2D beam-rocking case. The 2D pattern comprising a horizontal slice of the data cube is called the ionization-channeling pattern (ICP), which is used for the HARECXS or HARECES analysis and is the counterpart of an elemental map (EDX) or energy filtered image (EELS) for STEM spectral imaging.

Currently, ALCHEMI or HARECXS is automatically conducted using a PC-controlled scanning transmission electron microscope. In the beam alignment procedure for 2D beam-rocking ALCHEMI, the alignment of the condenser lens deflector is critical for the electron probe to stay at the same position on the sample during the beam tilt. Unfortunately, it is not possible to perfectly fix the beam pivot point during beam rocking because of the intrinsic aberrations of the microscope lens system, and the beam displacement with respect to the pivot point should be hopefully suppressed to no larger than one-tenth of the beam diameter. In general, the larger the tilt angle, the more significant is the pivot point fluctuation. Additional corrections are required for analyzing a smaller area using a smaller probe size. For HARECES, the diffraction pattern shift associated with the beam tilt should also be compensated for, which is described in detail below.

### 2.2. Calibration of probe shift associated with beam tilt

Next, we adopted software control of the electron probe for the present purposes instead of modifying the microscope hardware, because it was easier, more cost effective and machine independent. We thus took advantage of *QED* (Koch, 2011 ▸). This helps users to acquire large-angle rocking-beam electron diffraction and precession electron diffraction patterns from nano-sized samples by controlling the tilt angle and position of a collimated electron beam, thereby compensating for the beam shift induced by the aberrations of the illumination system.

In order to compensate for this probe shift, *QED* measures the aberrations of the illumination system and calibrates the corresponding deflectors. In particular, when the incident beam is tilted using the beam-tilt deflector, the diffraction on the back focal plane of the objective lens moves. *QED* compensates for this diffraction movement using the image shift (or diffraction shift) deflector (the operation is called descan), which is critically important for HARECES (EELS) measurements.

**Figure 2:**
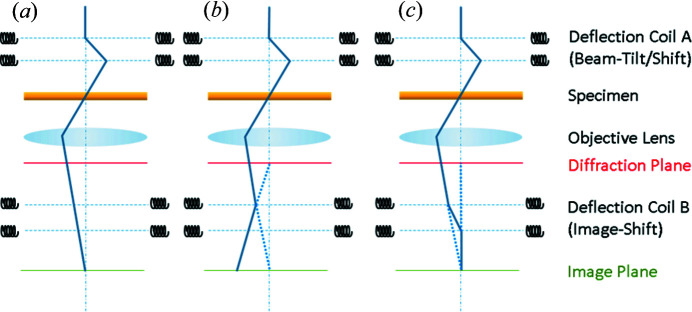
Ray diagrams for beam-tilt and descan operations. (*a*) Beam tilt with no descan, where the image does not move. (*b*) Descan with single-step deflection, so that the diffraction pattern is shifted back, where the image moves substantially. (*c*) Descan with double-step deflection, where the diffraction pattern is shifted back without image movement.

For a post-column type of electron energy-loss (EEL) spectrometer, the present descan operation may give rise to the displacement of the entire EEL spectrum on the projector lens focal plane, as shown in Fig. 2 ▸, where the objective lens pre-field, the primary origin of the aberrations causing the probe pivot-point shift, is deliberately not shown for simplicity. This undesirable spectral shift occurs for single-step beam deflection using a single deflector coil (Image Shift 1 or Projector Lens Alignment in JEOL transmission electron microscopes) alone [Fig. 2 ▸(*b*)], whereas a ‘diffraction shift’ (adopted in the current FEI scanning transmission electron microscopes), which corrects the shift with a two-step deflection using both image deflector coils, causes no such problem [Fig. 2 ▸(*c*)]. This spectral shift is usually small enough to allow one to neglect the resulting spectral intensity change for a maximum beam tilt less than ± a few degrees, although whether the effect will be significant highly depends on the hardware system and experimental conditions used.

### 2.3. EDX and EELS acquisition in ALCHEMI mode

*QED* was originally designed to acquire convergent beam diffraction patterns (Tanaka *et al.*, 1980 ▸) using a CCD/CMOS camera with tilting of the beam: as standard *QED* functions, large-angle rocking-beam electron diffraction, precession electron diffraction, diffraction mapping and fluctuation electron microscopy are implemented (HREM Research Inc., 2011 ▸; Koch, 2011 ▸). For *QED* to realize EEL and EDX spectral acquisition synchronized with incident-beam rocking, we have developed a script called the ‘ALCHEMI’ mode in *QED*, which enables *QED* to execute any custom *DM* script with the probe tilted instead of camera recording, allowing us to acquire either EDX or EELS spectra or both simultaneously for HARCEXS and HARECES analysis. The architecture of the ALCHEMI mode is described in the supporting information S1.

The ALCHEMI mode repeats the set of spectral acquisitions (from cueing the single or multiple spectral acquisitions to transferring the data to the buffer memory) in the *DM* script, before returning to QED’s main process for the next beam-tilt step. For concurrent EDX and EELS spectral acquisitions, ‘threads’ can be utilized, wherein the script for EDX, written in a separate thread, is executed in the background while the other is running. Flow charts of the present ALCHEMI mode and the scripts used in the present paper are also provided in the supporting information S1.

Since the spectral intensities of EDX and EELS exhibit very different yields for the same recording time, the thread for a single EDX acquisition at each beam tilt is executed in the background, while the required number of spectra for EELS are acquired within the specified time for a single shot. The set of EELS data at each beam tilt is stored as it stands, and each set can then be aligned and summed to correct the conceivable spectral energy drifts in a batch after the entire data acquisition is completed.

## 3. Experimental details

**Figure 3:**
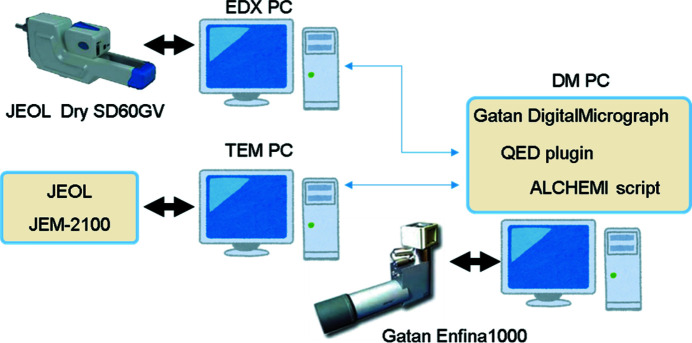
Present experimental setup for data acquisition.

A block diagram of the present hardware system to demonstrate the developed ALCHEMI mode is shown in Fig. 3 ▸. We used a JEOL JEM-2100, equipped with a Gatan Enfina 1000 for EELS acquisition and a JEOL silicon drift detector, Dry SD60GV, for EDX acquisition, which are controlled with three PCs, referred to as the TEM PC, DM PC and EDX PC, running on 32-bit Windows OS. The three PCs communicate with one another through *GMS* version 2.3 running on the DM PC, which controls EELS and image acquisitions (with a bottom-mount CCD camera, Gatan Orius SC200, and a wide-angle CCD camera, Gatan ES500W Erlangshen). *GMS* communicates with the TEM PC to control the probe motion and the EDX PC to start the EDX spectrum acquisition and subsequently transfer the data to the DM PC.

The present JEOL TEM/STEM control system has an attachment scanning image display (ASID) window, the STEM controller of the JEOL microscope to which we add the capability to change the incident beam direction in the TEM mode using the STEM scan signal received from the TEM PC. Thus, using the ASID, 2D HARECES data can be obtained by the conventional procedure to acquire a 2D scanning image. However, the electron illuminating area (∼1 µm ∅) fluctuates by ∼100 nm because of the lens aberrations as described above.

We conducted three experiments in this study. Firstly, the probe shift on the sample was evaluated to determine the smallest experimental area accessible by tilting the incident beam. The second experiment aimed to confirm if *QED* controlled the incident beam direction properly and gives 2D HARECXS data equivalent to those obtained with the ASID scheme. For this experiment Eu^3+^-doped Ca_2_SnO_4_ was selected as a test sample, where the dopant Eu^3+^ occupied both the Ca^2+^ and Sn^4+^ sites by nearly the same amount to keep charge neutrality (Fujimichi *et al.*, 2010 ▸). Finally, 1D HARECES was demonstrated for BaTiO_3_ (Ba and Ti/O planes are alternately stacked in the 〈100〉 direction) under the conditions where the 100 systematic rows of reflections were excited, additionally using the descan function to keep the diffraction boundary condition for each beam tilt.

## 4. Results and discussion

It is necessary to select an appropriate combination of the condenser aperture size, spot size and α-selector value (specific to the JEOL transmission electron microscope used) to ensure the smaller spatial resolution (probe size) and angular resolution (convergence angle) of the incident electron beam, with sufficient EDS/EELS signal intensities maintained as a trade-off.

### 4.1. Probe shift measurement in dark-field mode

In addition to the net probe position fluctuation due to the illuminating lens aberrations mentioned above, the aberrations of the objective lens induce a tilt-dependent image shift of the illuminating probe, thereby amplifying its actual movement on the sample. *QED* first calibrates the total image shift due to the instrumental aberrations for a wide range of beam tilts (HREM Research Inc., 2011 ▸; Koch, 2011 ▸). However, even after the standard beam calibration, a residual beam shift still remains because of the imperfect aberration calibration, which is particularly significant for smaller probes. The probe illuminating area on the sample is thus broadened compared with the specified probe size, and it is necessary to evaluate the amount of residual beam shift, which can be a critical parameter for smaller probe settings. For this purpose, the same experimental procedure as the beam calibration in *QED* can be utilized. The simpler method (*QED* has two modes, bright-field mode and dark-field mode) is described in the supporting information S2, where an incident electron beam is focused on a thick amorphous carbon film, and the probe image is observed by inserting an objective aperture on the optic axis (dark-field mode).

**Figure 4:**
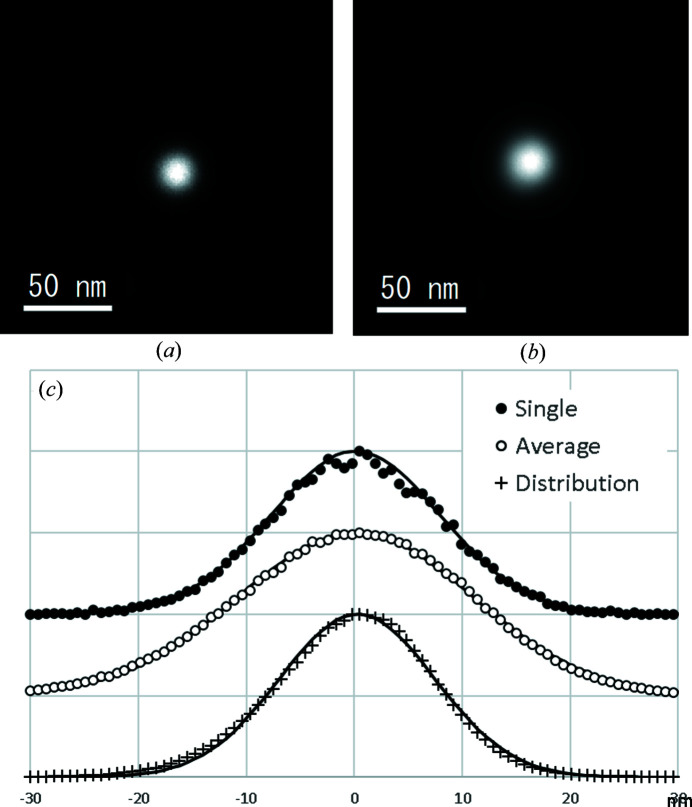
Probe movement analysis. (*a*) Single probe image without beam tilt. (*b*) Probe image averaged over the entire tilt range. (*c*) Beam profiles of the single and averaged probe images together with the probe distribution, estimated by numerical deconvolution.

To evaluate the net probe position fluctuation after the standard *QED* calibration procedures in the present system, the smallest probe condition practically feasible for HARECXS analysis was selected, where the spot size was set to ‘5’ (smallest), the α selector was set to ‘1’ (minimum convergence), and the third-largest condenser aperture (50 µm ∅; convergence semi-angle = 3.3 mrad) was used for ensuring a sufficient incident beam intensity and angular resolution. The probe shift on the sample was measured in the image mode while 2D-tilting the probe over a 60 mrad range (9 × 9 tilts) with a step of 7.5 mrad. Fig. 4 ▸(*a*) shows the probe image in the initial direction without a beam tilt, while Fig. 4 ▸(*b*) shows the probe image averaged over all the beam-tilt directions. These intensity profiles were fitted by Gaussians of FWHM 18.5 and 25.7 nm, respectively, as shown in Fig. 4 ▸(*c*). The average probe image was numerically deconvoluted with the single probe image to evaluate the probe shift distribution to be Gaussian with an FWHM of 16.84 nm, as also shown in Fig. 4 ▸(*c*). This suggests that the beam shift can be ascribed to a random fluctuation of the probe pivot point, rather than systematic errors caused by the *QED* beam control and calibration. The minimum area to be analyzed is hence estimated to be approximately 30 nm ∅ with the present STEM–*QED* combination. In Tables S1 and S2 in the supporting information S3, the relations between the spot size and probe diameter, and between the condenser aperture and convergence angle, for the present TEM system are reported, respectively. Users can select appropriate combinations depending on the experimental conditions engaged.

### 4.2. Comparison between HARECXS data obtained with ASID and *QED*

For this experiment we prepared a Ca_2_SnO_4_ grain oriented with the [201] zone along the incident beam direction, where the Ca and Sn columns are spatially separated in the projection. The smallest condenser aperture (10 µm ∅) was used to provide a convergence semi-angle of 0.63 mrad, similar to that with ASID hardware control for comparison, where the spot size was set to be ‘1’ (probe size approximately 144 nm) to ensure sufficient EDS counts within a reasonable time.

**Figure 5:**
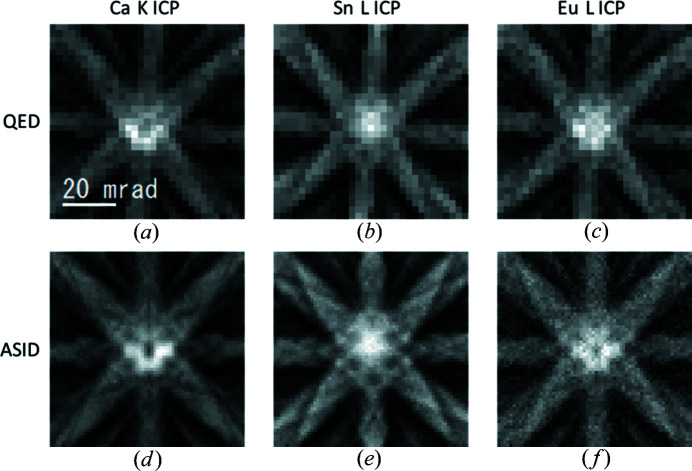
EDX ICPs obtained from Ca_2_SnO_4_. (*a*)–(*c*) Ca *K*, Sn *L* and Eu *L* ICPs obtained with *QED*, respectively. (*d*)–(*f*) Ca *K*, Sn *L* and Eu *L* ICPs obtained with the ASID, respectively.

Figs. 5 ▸(*a*)–(5 ▸ *c*) show HARECXS ICPs of Ca *K*, Sn *L* and Eu *L*, respectively, collected with *QED* software control. Each ICP consists of 31 × 31 spectra obtained by tilting the probe over ±35 mrad in the *x*- and *y*-axis directions around the [201] zone axis with a step of 2.3 mrad. Here, the spectral acquisition time for each tilt was 1 s, which implies that the total spectral acquisition time would be approximately 16 min for each frame. However, in practice, the total spectral acquisition time for each frame was approximately 30 min owing to some other extra controls. To obtain 3D HARECXS data with appropriate signal intensity, we acquired eight frames.

For comparison, a similar data set with the conventional ASID hardware control is shown in Figs. 5 ▸(*d*)–5 ▸(*f*), collected from the same crystal grain using a probe of approximately 1 µm ∅ because of system limitations (Muto & Ohtsuka, 2017 ▸). Here, a set of 64 × 64 spectra was collected at 1 s per frame and accumulated until the total spectral counts per pixel reached a level similar to that of the *QED* acquisition.

The statistical ALCHEMI analysis protocol based on the linear regression was applied to the data sets acquired by *QED* and ASID, which provides nearly the same Eu occupancies at the Ca site within the experimental accuracies of 58.2 ± 1.17% (*QED*) and 58.8 ± 0.68% (ASID), respectively.

Note that the *xy* coordinates in the present *QED* scheme are referred to the camera coordinate system, whereas those in the ASID scheme can be freely rotated by controlling the scan direction. In Figs. 5 ▸(*d*)–5 ▸(*f*) (ASID scheme), the scan direction was rotated to match the ICP coordinates to the horizontal and vertical axes of the display window. On the other hand, the ICPs of *QED* [Fig. 5 ▸(*a*)–5 ▸(*c*)] are rotated by approximately 5° owing to the actual sample orientation.

### 4.3. Automatic HARECES acquisition based on the *QED* scheme

**Figure 6:**
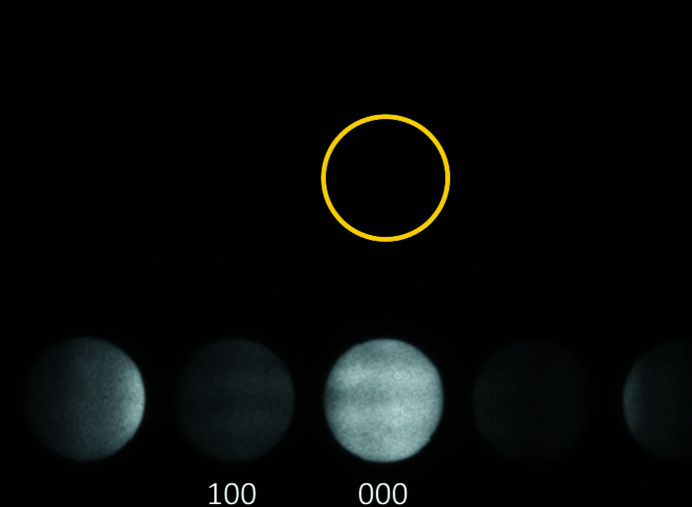
Diffraction pattern of the 100 systematic rows of reflections for BaTiO_3_, where the position of the EELS entrance aperture is shown by a yellow circle with a radius corresponding to the experimental semi-angle 2.76 mrad.

ALCHEMI–EELS or HARECES data were acquired for BaTiO_3_ by tilting the incident beam direction so that the EELS entrance aperture was always kept on the line that passes through the transmitted beam (000 spot) perpendicular to the 100 systematic rows of reflections (Tatsumi & Muto, 2009 ▸; Muto & Ohtsuka, 2017 ▸), as shown in Fig. 6 ▸. A set of 50 spectra were collected by tilting the beam from the condition with **K** = −2**g** to **K** = +2**g**, where **g** = 100, and **K** is the two-dimensional wavevector of incident electrons projected onto the *xy* plane. All the acquisition procedures were automatically controlled through the *QED* scheme with the descan mode on. Previously, HARECES experiments used to be conducted with the EELS aperture position manually adjusted for each beam tilt. The EELS entrance aperture was placed at 1.5**g** away from the 000 spot in the [010] direction, and the collection semi-angle subtended by the EELS entrance aperture was 2.8 mrad, which balances the trade-off between the spectral signal-to-noise ratio and better localization of the electron trajectories to the atomic columns (Taftø & Krivanek, 1982 ▸; Tatsumi & Muto, 2009 ▸).

In the present HARECES experiment the third-largest condenser aperture (‘3’; 50 µm ∅) was adopted and the spot size was set to be ‘3’, giving a probe size of 61 nm and a convergence semi-angle of 2.8 mrad. This again ensures the balance between the probe size and electron dose.

**Figure 7:**
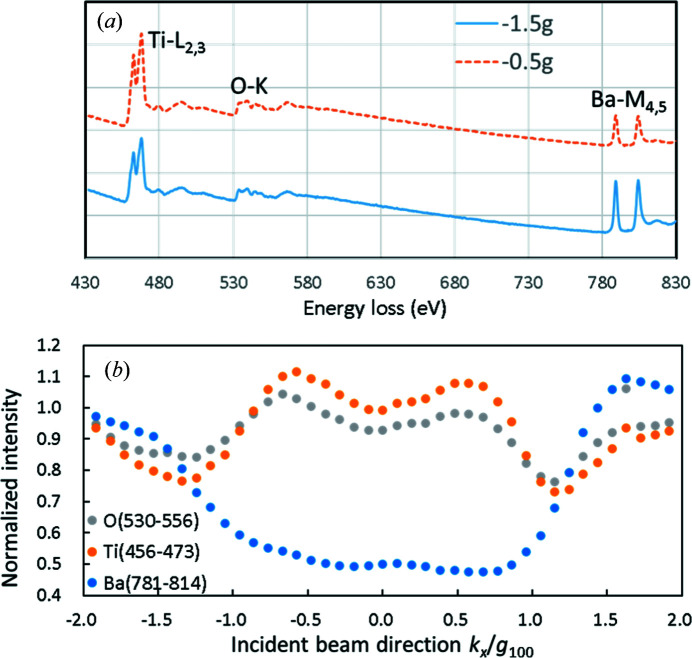
(*a*) EEL spectra of BaTiO_3_ taken at the beam-tilt angle corresponding to the scattering vectors **K** = −1.5**g** and **K** = −0.5**g**. The vertical axis represents the spectral counts, but the two spectra are shifted with respect to each other for better comparison. (*b*) Experimental core-loss signals of the O *K*, Ti *L*_2_,_3_ and Ba *M*_4,5_ edges integrated over the indicated energy ranges as functions of the beam direction from −2**g** to +2**g**. Each data set is normalized by the value at *k_x_*/*g*_100_ = −2.0.

The two EEL spectra taken at the tilt angles corresponding to **K** = −1.5**g** and **K** = −0.5**g** are shown in Fig. 7 ▸(*a*), and the integrated net core-loss signals of the Ti *L*_2,3_, Ba *M*_45_ and O *K* edges are shown in Fig. 7 ▸(*b*) as a function of the beam-tilt angle. The figures clearly demonstrate the electron-channeling effects, where the spectral intensities from the Ti/O planes and the Ba planes exhibit the steepest changes around **K** = −1.0**g** in the opposite manner, suggesting that the correct site-specific dependence of the core-loss spectral intensities of the constituent elements can be obtained even from an area as small as approximately 60 nm. The slight asymmetry in the curves in Fig. 7 ▸(*b*) can be attributed to the misorientation of the sample associated with local bending.

## 5. Summary

We have developed a software-control system that allows us to access ALCHEMI or HARECXS/HARECES measurements from a sub-micromtre area by automatically and concurrently tilting the beam and acquiring spectra using *QED*, a commercial software package controlling the electron probe and recording the diffraction or image with cameras for various advanced electron diffraction studies. The probe position movement due to the lens aberration of the illumination system was successfully suppressed using the ALCHEMI mode of *QED*. The present scheme thus overcomes the limitation of the analyzed area being no smaller than 1 µm ∅ and reduces the minimum size to as small as approximately 30 nm in diameter. As an application example of the present scheme, it was confirmed that *QED*–HARECXS provides the same accuracy as the conventional ASID hardware control. Moreover, HARECES was successfully performed by automatically canceling the diffraction shift using the descan option in *QED*, which has not been realized in the conventional ASID scheme. As the present software scheme runs on *GMS*, automatic ALCHEMI in principle can be executed on any type of digitized transmission electron microscope in which *QED* works. It is possible to evaluate whether *QED* works on the system by running ‘QED test…’ in the pull-down menu of *QED*.

Note that the FEI-manufactured transmission electron microscopes can be fully controlled by the unified open-source software platform called *TIA*, by which means the software for automatic HARECXS/HARECES scheme can be independently developed (Yamamoto *et al.*, 2016 ▸, 2018 ▸).

## Supplementary Material

Supporting information file. DOI: 10.1107/S1600576722003818/jl5035sup1.pdf
