# Supplementary material for: Automating ALCHEMI at the nano-scale using software compatible with PC-controlled transmission electron microscopy
Source: J Appl Crystallogr. 2022 May 25;55(Pt 3):551–7. doi: 10.1107/S1600576722003818 (PMC9172042; doi:10.1107/S1600576722003818)
Supplement: Supplementary file 1 [file j-55-00551-sup1.pdf]

JOURNAL OF
APPLIED
CRYSTALLOGRAPHY

Volume 55 (2022)

Supporting information for article:

Automating ALCHEMI at the nano-scale using software compatible with the current PC-controlled transmission electron microscopy

Akimitsu Ishizuka, Masahiro Ohtsuka and Shunsuke Muto

S1. ALCHEMI mode of QED

S1.1. Operation procedure for 1D-HARECES [S1, S2]

The QED plug-in enables us to access specific diffraction information without modifying the TEM hardware, one of the operation windows of which is shown in Fig. S1(*a*) as an example. Upon request by users, the ALCHEMI mode is implemented as one of the tabs in the QED Acquisition Tool window, as shown in Fig. S1(*b*). Unlike the acquisition tools originally implemented, such as LARBED/PED and DiffMap/FEM tabs (Fig. S1(a)), in the ALCHEMI mode the user must first specify the output image size (Frame size) for multiple spectral acquisitions, and the 'line' radio button can be selected as a scan pattern for conducting 1D-HARECES measurement using EELS, as described in Section 4.3 of the main text.

First specify the diffraction conditions for the initial and final tilt angles in the diffraction plane by pressing the <Set Start> and <Set Stop> buttons in the ALCHEMI line scan for each setup. Frame size should be set according to the pixel size of the detector employed. In the present study, the frame size is specified in the user-defined function (*cf*, next section S1.2) and the frame size should be identical to the specified values therein. The <Set Reference > button memorizes the original diffraction condition on clicking the button, which is reproduced by pressing the <Reset Tilt> button. This function is convenient for the user not to get lost in the initial diffraction condition when the user wants to modify the <Set Start> and/or <Set Stop> conditions after the measurement.

It is noted that both the <Descan> and <Non-linear Descan> checkboxes should be checked for HARECES because the diffraction spot shift associated with each beam tilt must be more precisely compensated for to strictly keep the detector position fixed with respect to the transmitted beam spot. For this purpose, execute <Calibrate Non-Linear Descan> in the Calibration tab prior to the ALCHEMI mode setting with the <Compensate Illumination Aberrations> check box on. It is necessary to use a high-resolution camera rather than a wide-range low-resolution camera for this operation.

Finally, press <Acquire ALCHEMI stack> to start the data acquisition procedure, which calls the user-defined "QEDAcquireALCHEMI" function, which is provided as a DM script file (*.s) and is registered in GMS in advance by selecting "Install Script File..." in the pull-down menu of the GMS "File" menu. The set of operations is schematically shown in the flow-chart in Fig. S2(*a*), where <Adjust probe position> keeps the probe position at the ROI on the sample using the pre-sample double deflection coil (*cf.* Coil A in Fig. 2). <Apply (non-linear) descan> keeps the detector position with respect to the transmitted beam on the diffraction plane for HARECES, using either Image Shift 1(JEOL TEMs) or post-sample deflection coils (FEI TEMs) (*cf.* Coil B in Fig. 2).

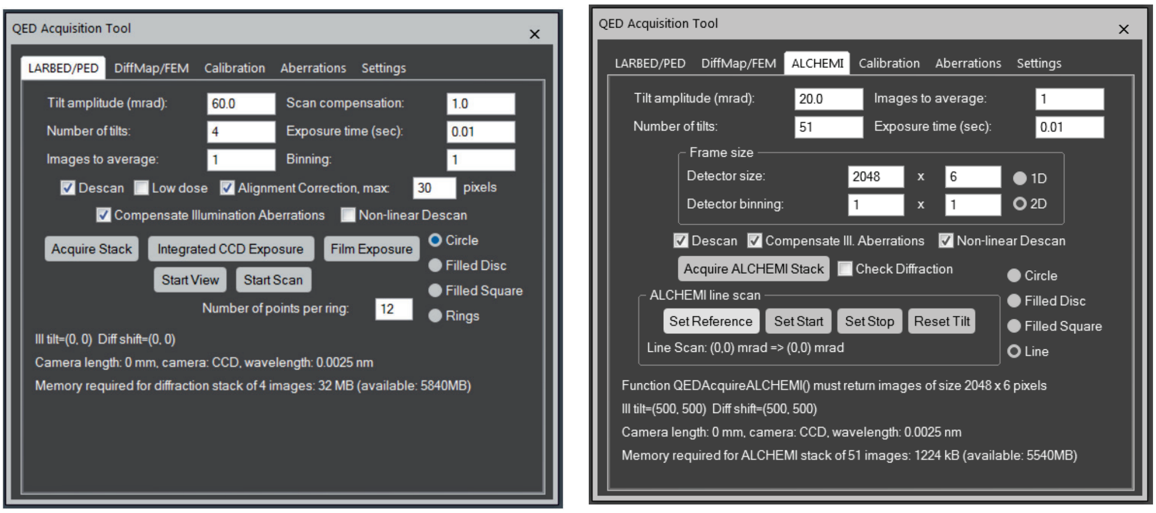

(a) (b)

Figure S1 Screen shots of the QED Acquisition tool windows: (a) Original version. (b) ALCHEMI mode implemented as an additional tab.

S1.2. Brief explanation of "QEDAcquireALCHEMI" script and 2D-HARECXS and concurrent HAREXCS/HARECES operations

The flow chart to illustrate how the "QEDAcquireALCHEMI" script generally works in the framework of the QED is shown in Figure S2 (*b*). 2D-HARECXS or concurrent HARECXS/HARECES can be done in the manner similar to the procedure above, by setting the scan pattern as "Filled Square" for 2D-HARECXS or selecting "Line" as the scan pattern.

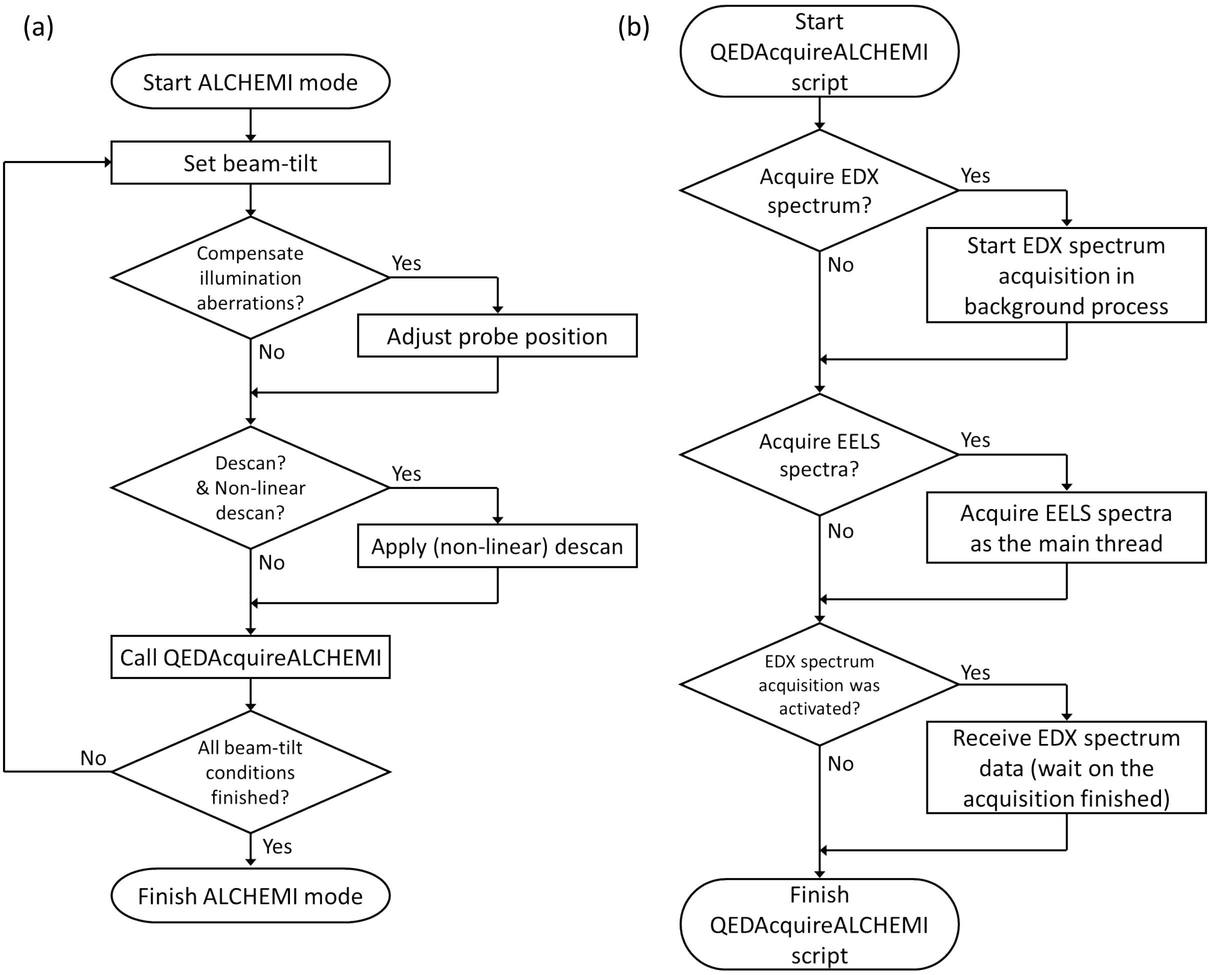

Figure S2 (a) Flow chart for ALCHEMI mode of QED. A house made function named 'QEDAcquireALCHEMI' is executed for each incident beam direction. QED corrects the probe displacement due to aberrations of the illumination system using the beam shift. For HARECES the diffraction pattern shifted with the beam tilt should be cancelled (descan). (b) Flow chart for scripts execution inside 'QEDAcquireALCHEMI'.

The following DM scripts, S−1, S−2 and S−3, are used for the HARECXS and HARECES experiments in Section 4.2 and 4.3 of the main text, working in the ALCHEMI mode. In the ALCHEMI mode, the function registered as a library with the fixed name, "QEDAcquireALCHEMI" (Script S−1), is called for each incident beam direction, the spectrum acquisition function for EELS, EDX or both is called inside the "QEDAcquireALCHEMI" for HARECXS, HARECES or concurrent HARECXS/HARECES, as shown in Fig. S1(*b*). The DM built-in commands "EDSAcquireSpectrum" and "EELSAcquireSpectrum" can be called for the EDX and EELS spectrum acquisitions, respectively through the bespoke scripts "ALCEDSAcqThread" (Script S-2) and "ALCEELSAcq" (Script S−3) inside "QEDAcquireALCHEMI". For concurrent HARECXS/HARECES with multi-thread programming, we have prepared the script "ALCEDSAcqThread" to run

“EDSSpectrumAcquire” in the background and the script “ALCEELSAcq” to run “EELSSpectrumAcquire” as the main thread. In the present “QEDAcquireALCHEMI”, first start the EDS acquisition in the background by calling the “ALCEDSAcqThread” class, followed by the EELS acquisition in the main foreground thread by calling “ALCEELSAcq”.

The “QEDAcquireALCHEMI” command in the script S-1 acquires the nEds sets of EDX (nEds 0 ∨ 1) and EELS spectra (nEels ≥ 0) and returns the acquired data as a 2D data array consisting of EDX and EELS spectra stored in order along the *y*-direction. The frame size should be set as 2D with a detector size of max(nChEds, nChEels) × Eds(nEds + nEels) pixels, where nChEds and nChEels refer to the numbers of EDX and EELS detector channels, respectively.

Script S-1. QEDAcquireALCHEMI (registered ALCHEMI function)

```
Void QEDAcquireALCHEMI(Image img){
  Number w, h;
  img.GetSize(w, h);

  Number nEds = 1; // EDS acquisition is enabled (1) or disabled (otherwise)
  Number nChEds = 4096; // Number of EDS detector channels
  Number dispEds = 0.1; // Dispersion of EDS detector (in keV)
  Number acqEds = 10; // EDS acquisition time (in seconds)
  Number timeOut = 30; // Timeout time when EDS is not responding (in seconds)

  Number nEels = 10; // Number of EELS acquisitions (0 => EELS is disabled)
  Number acqEels = 1; // EELS acquisition time (in seconds)
  Number sumFrames= 1; // Number of summing frames in a single EELS acquisition
  Number specBin = 1; // Spectrum binning in EELS acquisition
  Number vertBin = 100; // Vertical binning in EELS acquisition
  Number processing = 2; // Post processing for acquired spectrum
                         // 0: Unprocessed, 1: Dark Subtracted, 2: Gain Normalized

  Object oEDSSignal = NewSignal(0);
  Object oCancelSignal = NewSignal(0);
  Object cEDSAcq;

  if(nEds == 1){
    cEdsAcq = alloc(ALCEDSAcqThread).init(oEDSSignal, nChEds, dispEds, acqEds);
```

```
    cEDSAcq.StartThread(); // Start EDS acquisition in background
  }

  if(nEels > 0){
    Image EelsStack := ALCEELSAcq(nEels, acqEels, sumFrames, \
                                  specBin, vertBin, processing); // Acquire EEL
spectra
    Number nChEels = EelsStack.ImageGetDimensionSize(0);
    img[0, 0, nEels, min(nChEels, w)] = EelsStack[0, 0, nEels, min(nChEels, w)];
  }

  if(nEds == 1){
    oEDSSignal.WaitOnsignal(timeOut, oCancelSignal);
    if(!cEDSAcq.IsAcquiring()){
      Image EdsSpec := cEDSAcq.GetEDSImage(); // Receive EDS spectrum data
      nChEds = EdsSpec.ImageGetDimensionSize(0);
      img[nEels, 0 , nEels + 1, min(nChEds, w)] = EdsSpec[0, 0, 1, min(nChEds, w)]
    }
    else{
      Result("EDS acquisition has been timed out.\n");
    }
  }
}
```

Script S-2. ALCEDSAcqThread (for EDS acquisition in the background)

```
Class ALCEDSAcqThread : thread
{
  Image _imgEDS;

  Object _oSignal;
  Number _isAcquiring, _nChannels, _disp, _acqTime;

  Object init(Object self, Object oSignal, \
              Number nChannels, Number disp, Number acqTime){
    _oSignal = oSignal
    _isAcquiring = 1;
    _nChannels = nChannels;
    _disp = disp;
```

```
    _acqTime = acqTime;
    return self;
  }

  Void RunThread(Object self){
    _imgEDS := EDSAcquireSpectrum(_nChannels, _disp, _acqTime);
    _isAcquiring = 0;
    _oSignal.SetSignal();
  }

  Number IsAcquiring(Object self){
    return _isAcquiring;
  }

  Image GetEDSImage(Object self){
    return _imgEDS;
  }
}
```

Script S-3. ALCEELSAcq (for EELS acquisition)

```
Image ALCEELSAcq(Number nCycle, Number acqTime, Number sumFrames, \
                 Number specBin, Number vertBin, Number processing){
  Image imgTemp, imgR;
  Number nChannels;
  imgTemp := EELSAcquireSpectrum(acqTime, sumFrames, specBin, vertBin,
processing);
  nChannels = imgTemp.ImageGetDimensionSize(0);
  imgR := realimage("", 4,  nChannels, nCycle);
  imgR[0, 0, 1, nChannels] = imgTemp;
  for(Number i = 1; i < nCycle; i++){
    imgTemp := EELSAcquireSpectrum(acqTime, sumFrames, specBin, vertBin,
processing);
    imgR[i, 0, i + 1, nChannels] = imgTemp;
  }
  return imgR;
}
```

S2. Calibration of pseudo-aberrations in dark-field mode [S1, S2]

The aberration calibration in QED can compensate for the tilt angle-dependent movement of a small illuminated area on the sample by tracking the position of the on-axis dark-field (DF) image on the camera, as implemented in QED as one of the options in ‘Measure Illumination Aberrations’ command. This approach requires an amorphous sample to collect a detectable signal at all tilt angles.

An amorphous sample area is set within the field of view, a small objective aperture is inserted at the microscope’s optic axis, and then the movement of the probe on the sample can be tracked directly by following the movement of the DF image of the probe on the sample, independent of the aberrations of the objective lens. The DF image is created by diffracted electrons that pass through the objective aperture on the optic axis in this scheme.

S3. Probe diameter and convergence semi-angle on our microscope

The measured probe diameters and illumination convergence semi-angles for different spot size # and condenser aperture # are tabulated in Tables S1 and S2, respectively.

Table S1 Relation between spot size and probe diameter (illuminated area on the sample)

Here, the condenser aperture and α-selector are fixed to “3”, and “1”, respectively.

Spot size #	Probe diameter (nm)
1	145
3	48
5	19

Table S2 Relation between condenser aperture # and convergence angle

Here, the spot size and α-selector are fixed to “1” and “1”, respectively.

Condenser aperture #	Convergence semi-angle (mrad)
3	2.8
4	0.63

[S1] Koch (2011). *Ultramicroscopy* **111**, 828–840.

[S2] HREM Research Inc. (2011). https://www.hremresearch.com/qed/.
